# Correction to: Population Pharmacokinetic Evaluation of Amikacin Liposome Inhalation Suspension in Patients with Treatment‑Refractory Nontuberculous Mycobacterial Lung Disease

**DOI:** 10.1007/s13318-021-00687-z

**Published:** 2021-04-19

**Authors:** Christopher M. Rubino, Nikolas J. Onufrak, Jakko van Ingen, David E. Griffith, Sujata M. Bhavnani, Dayton W. Yuen, Kevin C. Mange, Kevin L. Winthrop

**Affiliations:** 1 Institute for Clinical Pharmacodynamics, Inc, 242 Broadway, Schenectady, NY USA; 2 Department of Medical Microbiology, Radboudumc Center for Infectious Diseases, Radboud University Medical Center, Geert Grooteplein 10, 6525 GA Nijmegen, The Netherlands; 3 The University of Texas Health Science Center at Tyler, 11937 US Highway 271, Tyler, TX USA; 4 Present Address: National Jewish Health, 1400 Jackson St, Denver, CO 80206 USA; 5 Insmed Incorporated, 700 US-206, Bridgewater, NJ USA; 6 Oregon Health and Science University, 3375 SW Terwilliger Boulevard, Portland, OR USA

## Correction to: European Journal of Drug Metabolism and Pharmacokinetics (2021) 46:277–287 10.1007/s13318-020-00669-7

The original version of this article unfortunately contained a few errors. The correct information is given below:

**Page 281, 1st paragraph, 2nd sentence**, **which previously read:**

The serum samples in TR02-112 were clustered in two primary windows—0–4 h and 12–24 h postdose (Fig. 1).

Should read:

The serum samples in TR02-112 were clustered in two primary windows—0–4 h and 12–24 h postdose (Fig. 2).

Figure 2: The 24- and 48-hour labels on the x-axis in Figure 2 were transposed. The corrected version of Fig. 2 is given below:

**Figure 2:**
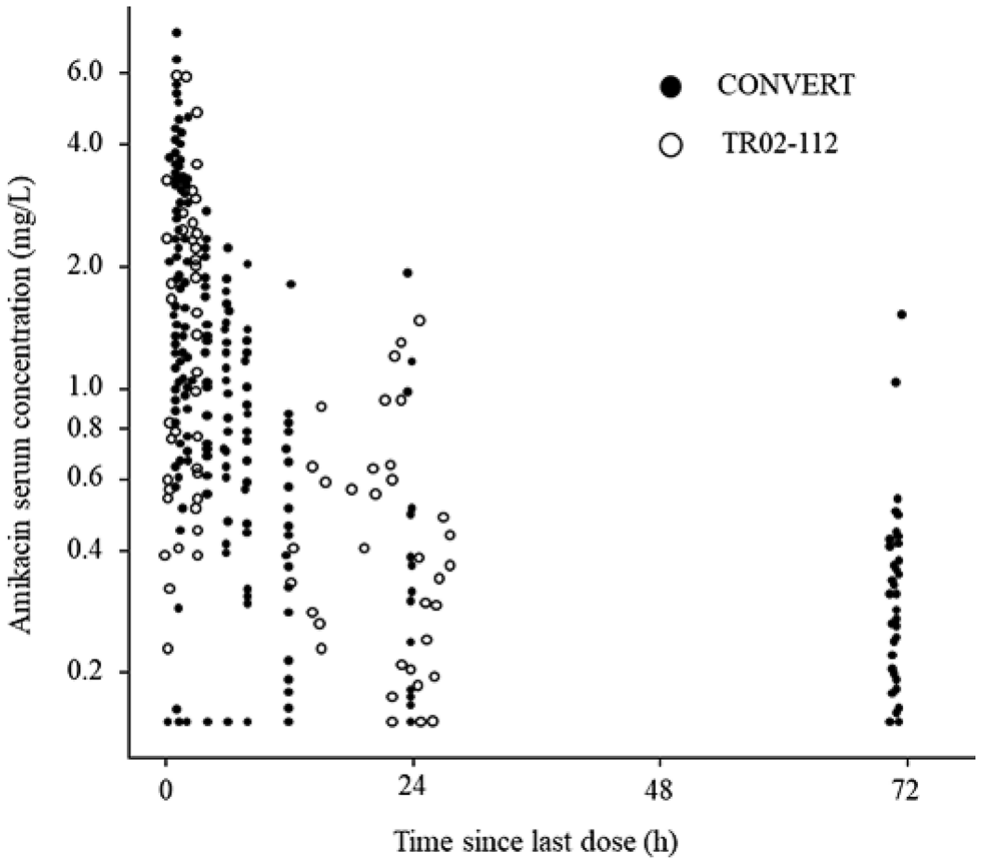
Observed serum amikacin concentration versus time sinces last dose. Closed and open circles represent individual values for patients in CONVERT and TR02-112 studies, respectively. Amikacin concentrations that were BLQ are shown at LLOQ (i.e., 0.15 mg/L). One BLQ concentration in the TR02-112 study, observed at > 500 h after the previous dose, was excluded; it was retained in the population pharmacokinetic dataset but did not impact the model fit. *BLQ* below the limit of quantification; *LLOQ* lower limit of quantification

The corrected version of supplementary file is updated here.

## Supplementary Information

Below is the link to the electronic supplementary material.

Supplementary file1 (PDF 405 kb)

