# Supplementary material for: Correction to: Population Pharmacokinetic Evaluation of Amikacin Liposome Inhalation Suspension in Patients with Treatment‑Refractory Nontuberculous Mycobacterial Lung Disease
Source: Eur J Drug Metab Pharmacokinet. 2021 Apr 19;46(4):573–4. doi: 10.1007/s13318-021-00687-z (PMC8298355; doi:10.1007/s13318-021-00687-z)
Supplement: Supplementary file 1 — Supplementary file1 (PDF 405 kb) [file 13318_2021_687_MOESM1_ESM.pdf]

European Journal of Drug Metabolism and Pharmacokinetics

ELECTRONIC SUPPLEMENTARY MATERIAL

Population Pharmacokinetic Evaluation of Amikacin Liposome Inhalation Suspension in Patients With Treatment-Refractory Nontuberculous Mycobacterial Lung Disease

Christopher M. Rubino, Nikolas J. Onufrak, Jakko van Ingen, David E. Griffith, Sujata M. Bhavnani,

Dayton W. Yuen, Kevin C. Mange, Kevin L. Winthrop

Corresponding Author: Christopher M. Rubino, crubino@icpd.com

Table of Contents

Measurement of amikacin concentration: sputum processing and bioanalysis. 2

Figure S1 Comparison of serum amikacin exposure estimates calculated using the population PK model and noncompartmental methods (comprehensive PK population; CONVERT).......... 3

Figure S2 Distributions of serum amikacin AUC_{0-24} and C_{max} estimates in Japanese and White patients.......... 4

Figure S3 Serum amikacin AUC_{0-24} after ALIS administration vs baseline lung (A) or renal (B) function.......... 5

Table S1 Sample collection schedule. 6

Table S2 Systemic bioavailability of amikacin after ALIS administration (TR02-112).......... 7

Measurement of amikacin concentration: sputum processing and bioanalysis

Amikacin was measured in serum and sputum samples using liquid chromatography with tandem mass spectrometry with an LLOQ of 0.15 mg/L for serum and 0.6 μg/g for sputum. Amikacin and the internal standard, gentamicin, were released from the matrix by protein precipitation. A similar process was used for urine with the exclusion of the protein precipitation step. After protein precipitation, the supernatant was diluted in the mobile phase and analyzed using reverse phase high-performance liquid chromatography. The analytical column was a BetaSil Phenyl 5B (100 × 2.1 mm). Amikacin and gentamicin were detected by monitoring the precursor and product ions (Rwz 586.4 + 425.2 for amikacin and Rwz 450.2 + 322.2 for gentamicin) using an Applied Biosystems AP13000 and AP14000 liquid chromatography with tandem mass spectrometry (LC/MS/MS) method. Serum, sputum/serum homogenate, and urine concentrations of amikacin were measured in 7 of the 9 clinical studies conducted with ALIS. In all studies, serum, sputum/serum homogenate, and LC/MS/MS method appropriate to the individual matrix at KCAS, LLC (formerly AAI Pharma), Shawnee, KS.

Starting in April 2013, a stable deuterium-labeled internal standard, Amikacin-d5, was used in place of gentamicin for the sputum/serum homogenate analysis by monitoring the precursor and product ions (m/z 591.4→ 430.2). The LLOQ was determined for serum (0.150 mg/mL), sputum (0.100 mg/mL), and urine (0.500 mg/mL).

Figure S1 Comparison of serum amikacin exposure estimates calculated using the population PK model and noncompartmental methods (comprehensive PK population; CONVERT)

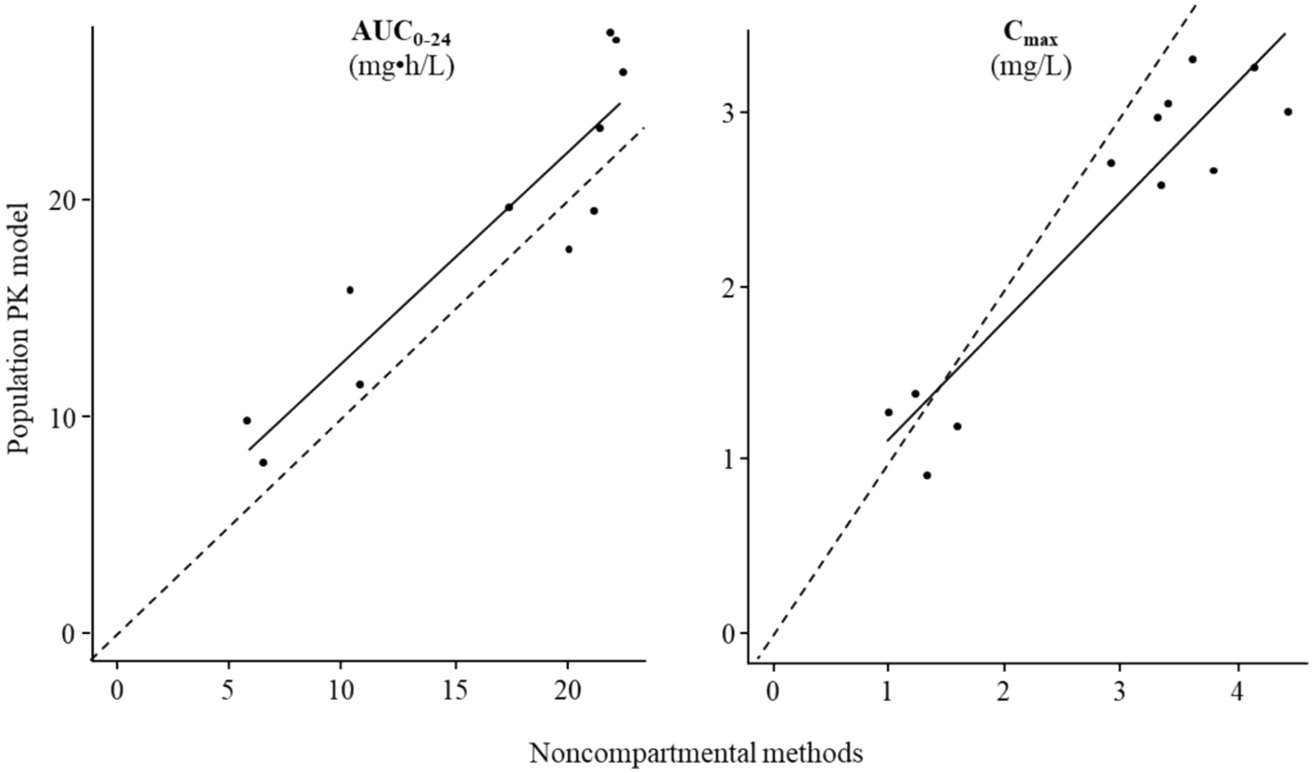

Each dot represents individual patient data. The solid line indicates the line of best fit, while the dashed line represents the line of identity. $AUC_{0\text{-}24}$, area under the plasma concentration time curve over a 24-h dosing interval; C_{max}, maximum concentration; PK, pharmacokinetic.

Figure S2 Distributions of serum amikacin $AUC_{0\text{-}24}$ and C_{max} estimates in Japanese and White patients

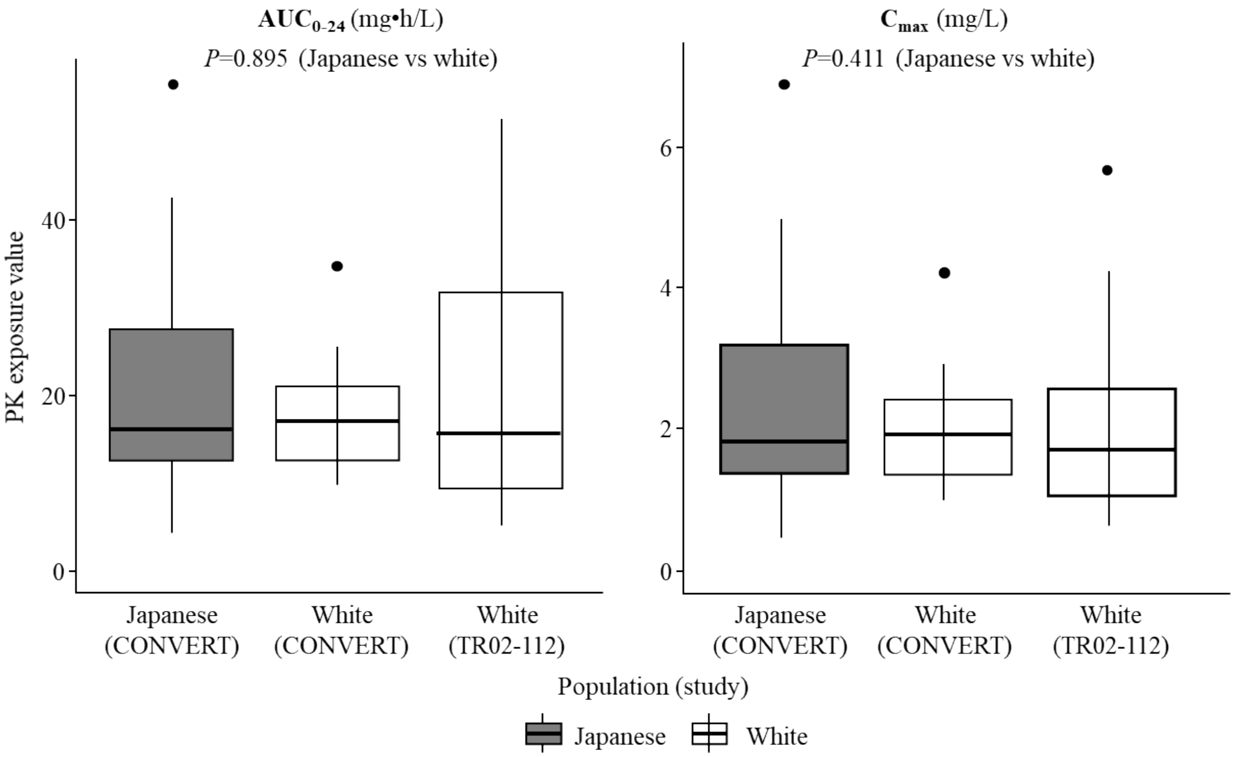

Each box represents the 25th to 75th percentile range, with median values shown by horizontal bars.

Whiskers show 1.5×interquartile range on each side, while black dots indicate values outside the whisker.

$AUC_{0\text{-}24}$, area under the plasma concentration time curve over a 24-h dosing interval; C_{max}, maximum concentration; PK, pharmacokinetic.

Figure S3 Serum amikacin $AUC_{0\text{-}24}$ after ALIS administration vs baseline lung (A) or renal (B) function

A. Lung

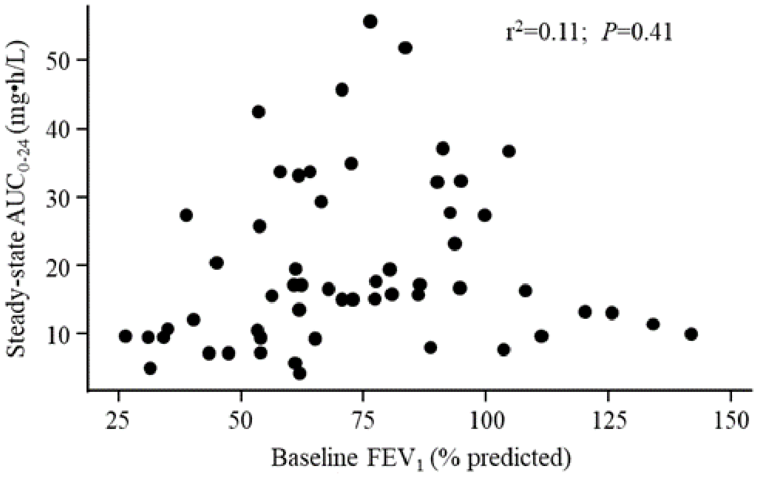

B. Renal Function

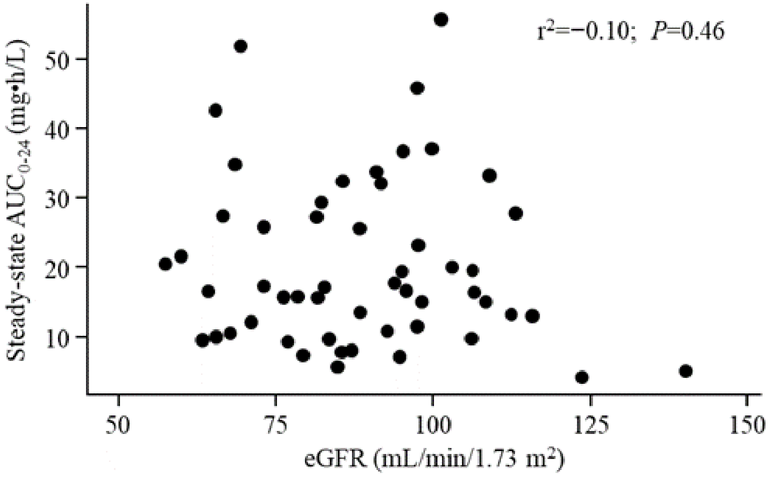

Each dot represents individual patient data. ALIS, amikacin liposome inhalation suspension; $AUC_{0\text{-}24}$, area under the plasma concentration time curve over a 24-h dosing interval; eGFR, estimated glomerular filtration rate; FEV_1, forced expiratory volume in 1 second; r^2 = coefficient of determination.

Table S1 Sample collection schedule

		TR02-112 (subset of White patients)							CONVERT									
									Population PK Subset						Comprehensive PK Substudy (Japanese pts)			
	Collection Time	Days							BL	Months					BL	Month		
		1	2	28	56	84	112	168	D1	1	3	4	5	6	D1	1	3	6
Blood	Predose 0-4 h	x	x	x	x	x	x	x										
	Postdose 0-4 h	x	x	x	x	x	x	x										
	Predose 0-1 h									x	x			x	x	x	x	x
	Postdose 1-4 h									x	x			x		x		x
	Postdose 1, 2, 4, 6, 8, 12, 24 h														x		x	
Sputum	Predose 0-4 h	x	x	x	x	x	x	x										
	Postdose 0-1 h	x	x	x	x	x	x	x										
	Predose 0-1 h									x[a]	x[a]			x[a]	x	x	x	x
	Postdose 1-4 h									x	x			x		x	x	x
	Postdose 8h, 24 h														x[c]			
	Predose/Postdose72 h								x[a,b]			x[a,b]	x[a,b]	x[a,b]				
Urine	Postdose 0-24 h	x				x		x										

BL, baseline; D, day; h, h; pts, patients.
[a] Samples collected after a 2-day dose interruption.
[b] Sputum-only subset; samples were also collected at off-treatment follow-up visits on day 28 and at 3 months; predose collection for BL only.
[c] Sampling at 8 h postdose was optional.

Table S2 Systemic bioavailability of amikacin after ALIS administration (TR02-112)

Day	% Dose excreted in urine
Day 1	
n	6
Mean (%CV)	4.46 (54.8)
Median (range)	3.25 (2.71-8.95)
Day 84	
n	6
Mean (%CV)	7.74 (77.5)
Median (range)	6.88 (1.55-17.2)
Day 168	
n	11
Mean (%CV)	8.85 (70.3)
Median (range)	8.42 (0.72-22.6)

ALIS, amikacin liposome inhalation suspension; CV, coefficient of variation.
